# Effects of batroxobin on the antithrombotic system in patients with cerebral venous thrombosis: Clues to mechanisms

**DOI:** 10.1111/cns.14861

**Published:** 2024-08-04

**Authors:** Duo Lan, Baolian Jiao, Siying Song, Mengqi Wang, Xiaoming Zhang, Xiangqian Huang, Yibing Guo, Yuchuan Ding, Xunming Ji, Ran Meng

**Affiliations:** ^1^ Department of Neurology, Xuanwu Hospital Capital Medical University Beijing China; ^2^ Department of China‐America Institute of Neuroscience, Xuanwu Hospital Capital Medical University Beijing China; ^3^ Division of Neurocritical Care and Emergency Neurology Massachusetts General Hospital, Harvard Medical School Boston Massachusetts USA; ^4^ Department of Neurosurgery Wayne State University School of Medicine Detroit Michigan USA

**Keywords:** antithrombus, batroxobin, coagulation, fibrinolysis, platelet aggregation

## Abstract

**Background and Purpose:**

More evidence supports the benefits of batroxobin combined with anticoagulation in correcting acute cerebral venous thrombosis (CVT). The dynamic fluctuations of peripheral blood platelets, fibrinolysis, and coagulation biomarkers during this therapy were analyzed.

**Methods:**

We investigated batroxobin's effects on the antithrombotic system under two regimens. The pretreatment group included patients on anticoagulants for at least 1 week before starting batroxobin. The simultaneous treatment group began both treatments upon admission. The control group received only anticoagulation. Batroxobin was given on alternate days at doses of 10BU, 5BU, and 5BU, totaling three doses. Anticoagulation was continuous. Baseline data were T0; the next day after each batroxobin dose was T1, T2, and T3. Data from these four time points was analyzed.

**Results:**

The time‐point paired sample T‐test results of the pretreatment group [n = 60; mean age (SD), 43.3(16.5); 38 (63.35%) women] showed that batroxobin significantly inhibited ADP‐induced platelet aggregation rate (T1–T0: *p* = 0.015; T2–T0: *p* = 0.025; T3–T0: *p* = 0.013), decreased fibrinogen level (T1–T0: *p* < 0.001; T2–T0: *p* < 0.001; T3–T0: *p* < 0.001), and increased D‐dimer (T1–T0:*p* < 0.001; T2–T0: *p* < 0.001; T3–T0: *p* < 0.001), TT (T1–T0:*p* = 0.046; T2–T0: *p* = 0.003; T3–T0: *p* < 0.001), and APTT (T1–T0:*p* = 0.021; T2–T0: *p* = 0.012; T3–T0: *p* = 0.026). Compared to the control group, the simultaneous treatment group showed significantly higher TT (T2: *p* = 0.002; T3: *p* = 0.004) and D‐dimer (T1: *p* < 0.001; T2: *p* < 0.001; T3: *p* < 0.001) values, while fibrinogen (T2: *p* < 0.001; T3: *p* < 0.001) levels were significantly lower. Using batroxobin can alleviate the amplitude of changes in coagulation indicators other than TT caused by anticoagulants. The above conclusions are consistent with the results of repeated measurement data analysis.

**Conclusions:**

Batroxobin can significantly inhibit ADP‐induced platelet aggregation rate, increase D‐dimer, decrease fibrinogen, and prolong TT and APTT in the presence of anticoagulant agents. Using batroxobin can reduce the amplitude of changes in coagulation indicators caused by anticoagulants. These results reveal the potential mechanism of batroxobin combined with anticoagulation in the safe and effective treatment of CVT.

## Graphical abstract

Batroxobin can inhibit platelet aggregation, degrade insoluble fibrinogen, dissolve soluble fibrin, and prolong TT and APTT which makes it play a stable role in the effective treatment of CVT. Batroxobin can slow down the changes in coagulation indicators, which makes it not increase the risk of adverse events, especially bleeding.

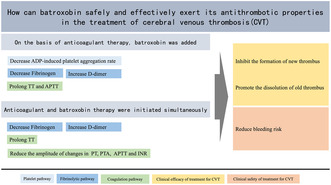

## 1 INTRODUCTION

Batroxobin, a thrombin‐like serine protease extracted from Bothrops atrox moojeni venom, has been shown to exert defibrinogenating effects by releasing fibrinopeptide‐A from fibrinogen and generating water‐soluble fibrin monomers.^1^ Moreover, batroxobin may induce endothelial cells to release tissue plasminogen activator (t‐PA) and reduce the activation of tissue plasminogen inhibitor (PAI), mimicking the thrombolytic mechanism where plasmin is generated from circulating plasma plasminogen by endogenous t‐PA.^2^ A growing body of evidence supported the benefits of batroxobin use in patients with cerebral venous thrombosis (CVT) and cerebral arterial thrombosis (CAT).^3^, ^4^, ^5^ However, reducing batroxobin‐related bleeding events, especially in patients concurrently undergoing anticoagulation, remains a significant challenge in treating cerebral thrombotic diseases.^6^, ^7^, ^8^, ^9^ Despite the known effects of batroxobin in significantly reducing fibrinogen levels while increasing D‐dimer,^10^, ^11^ almost no studies reported its impact on platelet and coagulant functions when combined with anticoagulation in treating CVT, except for our previous small case study,^5^ Yoshikawa et al. reported that batroxobin could prolong prothrombin time (PT) and activated partial thromboplastin time (APTT).^12^ However, Choi et al. and Ding et al. reported that batroxobin had no significant effects on PT and APTT, but could prolong thrombin time (TT).^13^, ^14^ These research findings on batroxobin's effect on coagulation indicators were inconsistent. These studies did not strictly regulate drug administration time, study indicator collection times, or patient medication use before enrollment, potentially contributing to the conflicting results. More importantly, no clinical study has investigated the impact of batroxobin on platelet function particularly when combined with anticoagulation, despite the known cornerstone role of anticoagulant therapy in CVT management.^15^

In this real‐world study, we explored the effects of batroxobin on platelet, fibrinolysis, and coagulation functions in CVT patients under different medication regimens to provide a basis for the safe and effective clinical application of batroxobin in CVT treatment.

## 2 METHODS

### 2.1 Study design and participants

Patients diagnosed with CVT from May 2017 through December 2022 at Xuanwu Hospital, Capital Medical University, were enrolled in this real‐world study. The diagnosis of CVT was confirmed according to AHA/ASA guidelines, utilizing magnetic resonance black blood thrombosis imaging (MRBBTI) and magnetic resonance venography (MRV), computed tomographic venography (CTV), or digital subtraction angiography (DSA).

The inclusion and exclusion criteria were as follows:

Inclusion criteria:

1. Patients with imaging‐confirmed CVT with a duration of illness of less than 1 month;
2. Age between 18 and 80 years;
3. Patients who received batroxobin and/or anticoagulant agent treatment post‐enrollment.

Exclusion criteria:

1. Patients who underwent antiplatelet therapy (e.g., aspirin), defibrinogenating therapy, and/or thrombolysis (e.g., recombinant tissue plasminogen activator, rt‐PA) before enrollment;
2. Patients with disorders affecting blood, coagulation, and immune system;
3. Patients with baseline plasma fibrinogen were equal to or less than 1 g/L;
4. Patients with incomplete data.

### 2.2 Intervention and assessment

Patients underwent batroxobin intravenous infusion after signing an informed consent. Batroxobin injection (produced by Beijing Tuobixi Pharmaceutical Co., Ltd., the approval number was H20031074) 10 BU for the first time, followed by 5 BU every other day unless the level of fibrinogen dropped to 1.0 g/L or less during treatments. Standard anticoagulation treatment in this study was defined as low molecular weight heparin (LMWH) subcutaneous injection (0.4 mg/q12h) for a total of 5–7 days, then bridged from LMWH to warfarin 3 mg/day orally.

We designed and investigated the effects of batroxobin on the antithrombotic system under two different medication regimens to better serve clinical practices and comprehensively evaluate batroxobin's overall effects through multi‐step and multi‐group analysis. In the first regimen, patients who had received long‐term anticoagulation therapy before admission and then added batroxobin treatment after admission were included. Patients who had been on anticoagulants for at least 1 week prior to starting batroxobin were categorized into the pre‐treatment group to explore the effects of batroxobin on the antithrombotic system in the context of long‐term anticoagulation. In the second regimen, patients simultaneously began anticoagulation and batroxobin treatment upon admission. These patients were included in the simultaneous treatment group to investigate the combined effects of batroxobin and anticoagulation on the antithrombotic system. Patients who received only anticoagulation therapy after admission were included in the control group to serve as a comparison for the simultaneous treatment group.

**FIGURE 1:**
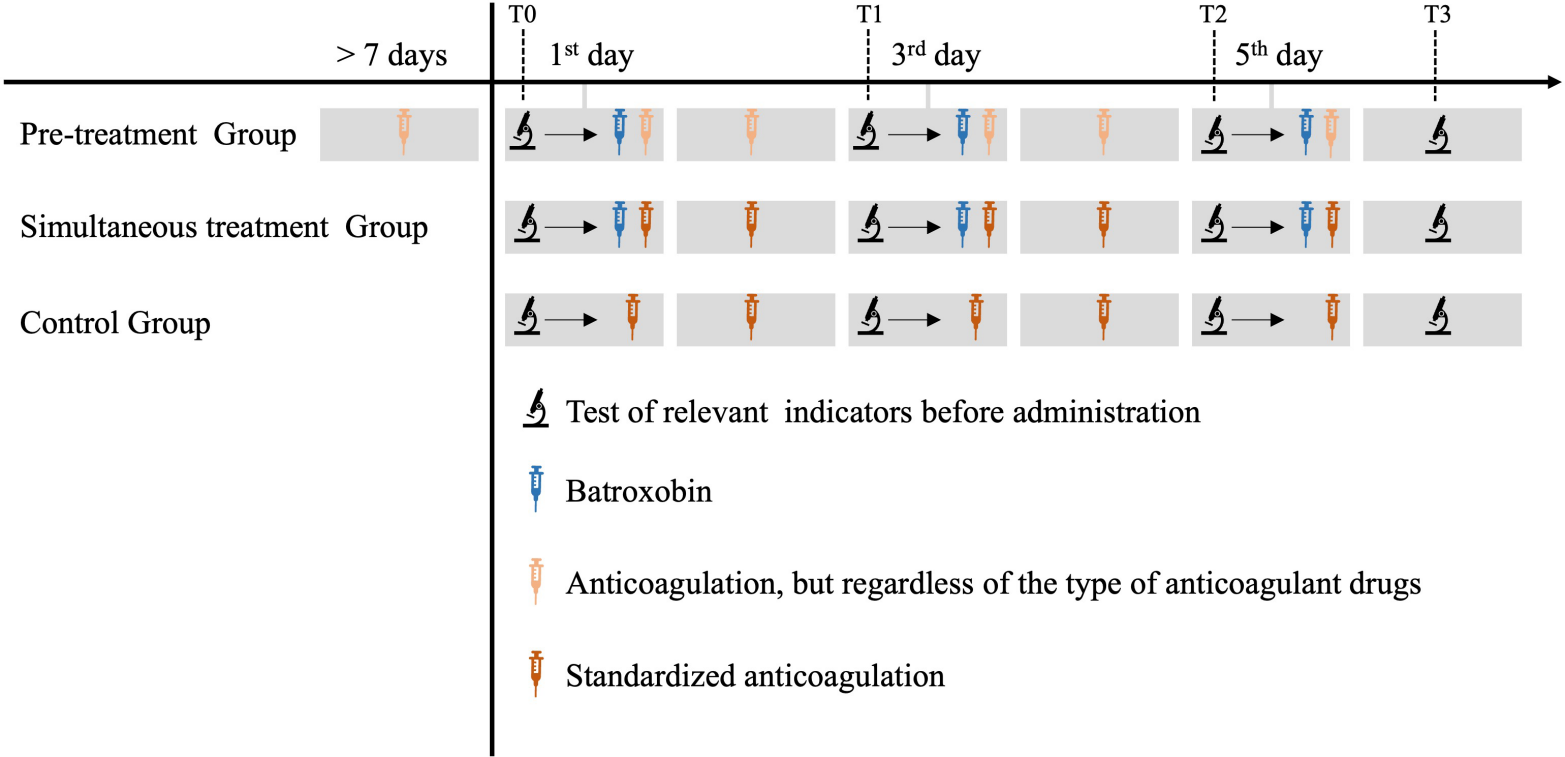
Clinical tests and treatment procedures for different groups and treatment regimes.

We collected data at four time points: before the first dosage of batroxobin (T0), before the second dosage of batroxobin (T1), before the third dosage of Batroxobin (T2), and after the third dosage of batroxobin (T3). The data collected included platelet‐related indicators (platelet counts [PLT], platelet crit [PCT], mean platelet volume [MPV], platelet distribution width [PDW], arachidonic acid‐induced platelet aggregation rate [AA‐induced induced platelet aggregation rate] and adenosine diphosphate‐induced platelet aggregation rate [ADP‐induced induced platelet aggregation rate]), coagulation indicators (activated partial thromboplastin time [APTT], prothrombin time [PT], thrombin time [TT], and prothrombin time activity [PTA]) and fibrinolytic indicators (fibrinogen and D‐dimer). The aforementioned indicators were re‐tested 24 hours after batroxobin and/or anticoagulation use and before the next dose use. The normal ranges were as follows: PLT (100–300 × 10^9^/L), PCT (0.1%–0.28%), MPV (9.4–12.5 fl), PDW (0–15%), AA‐induced induced platelet aggregation rate (52%–84%), ADP‐induced induced platelet aggregation rate (52%–84%), APTT (25–43.5 s), PT (11–15 s), TT (14–21 s), PTA (70%–120%), INR (0.8–1.2 INR), Fibrinogen (2–4 g/L), and D‐dimer (0.01–0.5 μg/mL). The outcomes were changes in these indicators at different time points and groups. Figure 1 details each group's specific drug and assay regimens and their corresponding time points.

### 2.3 Statistical analysis

We paired the data of the pre‐treatment group at each time point (T0–T1, T0–T2, T0–T3) and performed a paired sample T‐test to determine whether there were significant changes in antithrombotic indicators after three doses of batroxobin in the same patient. It is important to note that if the data at any time point in the paired dataset were missing, the patient's paired data were excluded from the analysis. Additionally, data from patients who received three doses of batroxobin and had complete data at all four time points was further subjected to repeated measures analysis of variance.

A comparative study was conducted between the simultaneous treatment and the control group. This comparison aimed to investigate whether there was a significant difference in the effect of batroxobin combined with anticoagulation versus anticoagulation alone on antithrombotic indicators. As described above, repeated measures analysis of variance and interaction analyses were performed on data from patients who received three doses of batroxobin and had complete data at all four time points.

Normally distributed continuous variables were presented as mean ± standard deviation (SD) and non‐normally distributed continuous variables as median (IQR). Categorical variables were described as numbers (percentages). The normality of distribution was visually tested with histograms and normal probability plots (Q‐Q plots). The Pearson chi‐square or Fisher's exact test was used to compare categorical data. Intergroup differences in continuous variables were tested via independent sample *t*‐test or Mann‐Whitney *U* test. Paired sample T test and repeated measurement data variance analysis and interaction effect analysis will be used in this study. *p* < 0.05 was considered indicative of statistical significance. R software 4.2.1 was used for data analysis.

## 3 RESULTS

Among the 90 patients, a total of 60 patients [mean age (SD), 43.3 (16.5); female, 38 (63.35%)] were enrolled in the pre‐treatment group. Of the remaining 30 patients, 15 patients [mean age (SD), 37.0 (15.5); female, 8 (53.3%)] were in the simultaneous treatment group, and another 15 patients [mean age (SD), 31.4 (10.3); female, 11 (73.3%)] were in the control group. The simultaneous treatment and the control group did not significantly differ in baseline demographic and risk factor characteristics. (Supplementary Table S1).

### 3.1 The effect of batroxobin on platelet indicators

**FIGURE 2:**
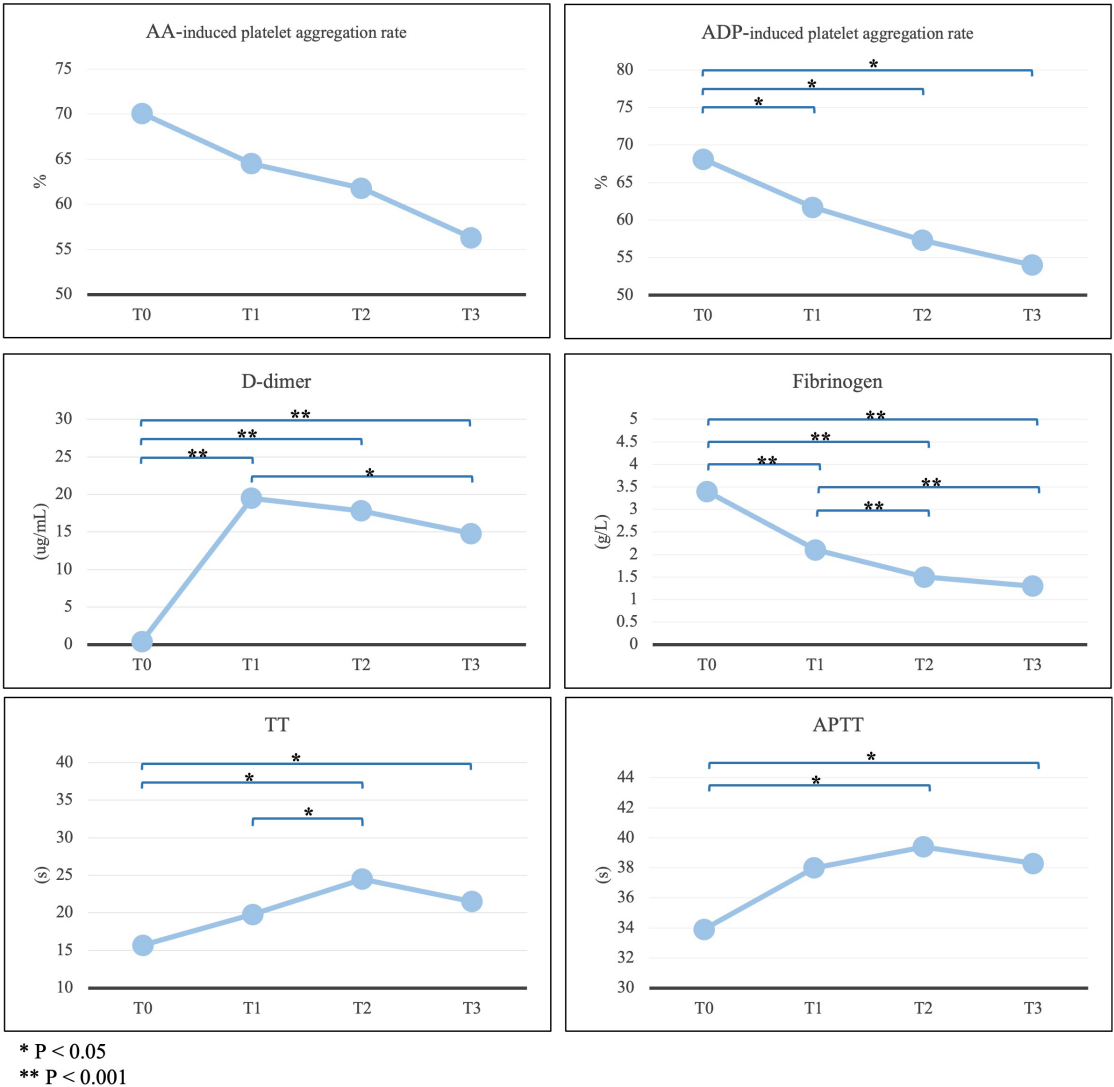
Repeated measurement data analysis results of antithrombotic indicators in the pre‐treatment group. APTT, Activated partial thromboplastin time; TT, Thrombin time.

The results of the time‐point paired T‐test showed that batroxobin had no significant effect on platelet indicators except ADP‐induced induced platelet aggregation rate in the pre‐treatment group. The values of ADP‐induced induced platelet aggregation rate decreased significantly after batroxobin use [T1–T0 mean difference (SD): −8.8 (15.7), *p* = 0.015; T2–T0: −13.7 (14.8), *p* = 0.025; T3–T0: −11.5 (13.3), *p* = 0.013] (Table 1). The significant difference was also found in the results of repeated measurement data analysis (*p* = 0.032) (Figure 2).

**TABLE 1:** Paired Sample T‐tests showing analysis results of antithrombotic indicators at paired time points in the pre‐treatment group.

	T1 vs. T0		T2 vs. T0		T3 vs. T0	
	Mean difference (SD)^a^	*p* Value	Mean difference (SD)^b^	*p* Value	Mean difference (SD)^c^	*p* Value
Platelet Indicators						
PLT (10^9^/L)	2.3 (31.2)	0.664	−2.2 (33.6)	0.755	3.2 (45.7)	0.808
PCT (%)	0.00 (0.03)	0.638	0.00 (0.03)	0.676	−0.02 (0.08)	0.384
MPV (fl)	0.1 (0.5)	0.138	0.1 (0.3)	0.348	−0.1 (0.3)	0.415
PDW (%)	0.1 (1.0)	0.425	0.3 (0.9)	0.186	−0.39 (0.7)	0.138
AA (%)	−6.3 (14.7)	0.059	−4.2 (14.3)	0.402	−5.2 (2.1)	0.073
ADP (%)	−8.8 (15.7)	0.015	−13.7 (14.8)	0.025	−11.5 (13.3)	0.013
Fibrinolysis indicators						
Fibrinogen (g/L)	−1.6 (1.0)	<0.001	−2.2 (1.4)	<0.001	−2.1 (0.9)	<0.001
D‐dimer (μg/mL)	18.7 (3.5)	<0.000	17.5 (4.9)	<0.001	14.6 (6.8)	<0.001
Coagulation indicators						
PT (s)	0.0 (0.8)	0.894	0.4 (0.09)	0.159	0.5 (5.2)	0.314
PTA (%)	−2.4 (11.4)	0.429	−5.8 (13.3)	0.109	−7.9 (13.3)	0.038
TT (s)	4.0 (7.1)	0.046	8.7 (9.3)	0.003	5.8 (5.2)	<0.001
APTT (s)	4.1 (6.1)	0.021	5.5 (7.3)	0.012	4.3 (6.7)	0.026
INR	0.0 (0.2)	0.486	0.0 (0.2)	0.979	0.0 (0.3)	0.796

*Note*: *p* value less than 0.05 is considered a significant difference. Abbreviations: AA, AA‐induced platelet aggregation rate; ADP, ADP‐induced platelet aggregation rate; APTT, Activated partial thromboplastin time; INR, International normalized ratio; MPV, Mean platelet volume; PCT, Platelet crit; PDW, Platelet volume distribution width; PLT, Platelet count; PT, Prothrombin time; PTA, Prothrombin time activity; TT, Thrombin time.

^a^ Mean difference of the T1 − T0.

^b^ Mean difference of the T2 − T0.

^c^ Mean difference of the T3 − T0.

**FIGURE 3:**
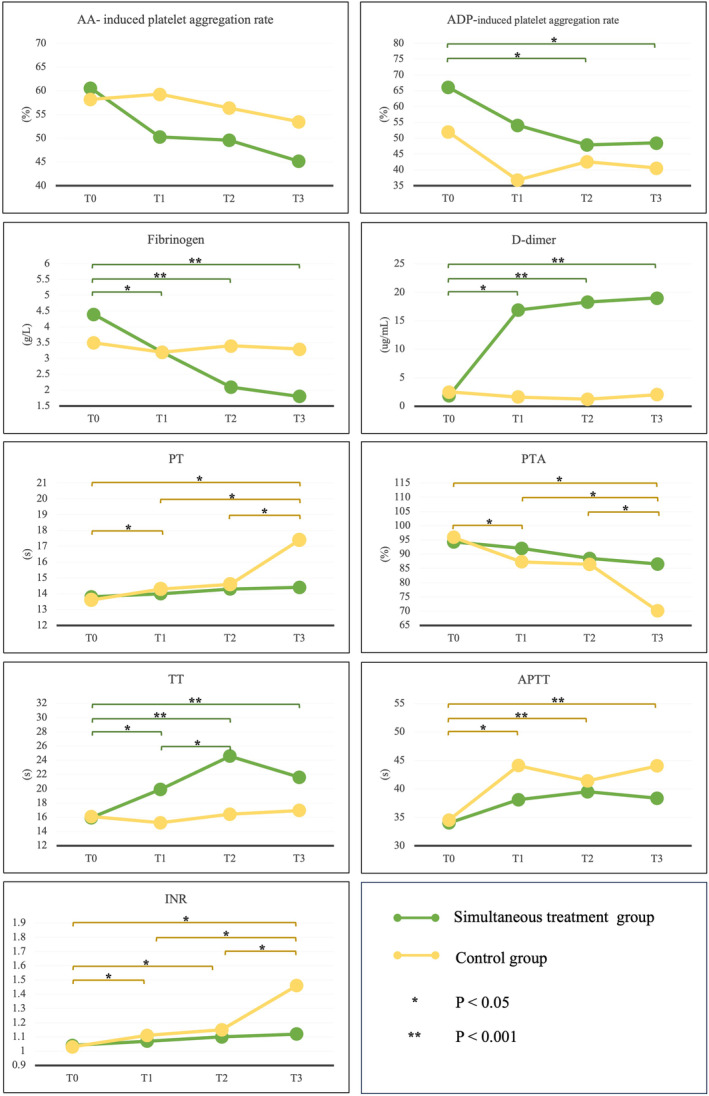
Repeated measurement data analysis results of antithrombotic indicators of the simultaneous treatment and the control group. APTT, Activated partial thromboplastin time; INR, International normalized ratio; PT, Prothrombin time; PTA, Prothrombin time activity; TT, Thrombin time.

A significant change was found in the results of repeated measurement and interaction effect analysis in the simultaneous treatment group. The values of ADP‐induced induced platelet aggregation rate (*p* < 0.05) at T0 were significantly different from the values at T2 and T3. (Figure 3).

### 3.2 The effect of batroxobin on fibrinolytic indicators

The results of the paired T‐test showed that batroxobin could significantly affect fibrinogen and D‐dimer values in the pre‐treatment group. The fibrinogen values significantly decreased after batroxobin use [T1–T0: −1.6 (1.0), *p* < 0.001; T2–T0: −2.2 (1.4), *p* < 0.001; T3‐T0: −2.1 (0.9), *p* < 0.001]. The D‐dimer values greatly increased after batroxobin use [T1–T0: 18.7 (3.5), *p* < 0.001; T2–T0: 17.5 (4.9), *p* < 0.001; T3–T0: 14.6 (6.8), *p* < 0.001] (Table 1). Significant differences were also found in the results of repeated measurement data and interaction effect analysis of fibrinogen (*p* < 0.001) and D‐dimer (*p* < 0.001). (Figure 2).

The simultaneous treatment group also observed significant effects of batroxobin on fibrinolytic indicators. Fibrinogen values were significantly decreased at T2 and T3 time points in the simultaneous treatment group compared with the control group. (T2 mean difference (SD): −1.3 (0.3), *p* < 0.001; T3: −1.5 (0.3), *p* < 0.001). D‐dimer values were significantly increased at T1, T2, and T3 time points in the simultaneous treatment group compared with the control group. (T1: 15.3 (1.6), *p* < 0.001; T2: 17.1 (1.2), *p* < 0.001; T3: 16.9 (1.3), *p* < 0.001). (Table 2) Significant changes were found in the results of repeated measurement and interaction effect analysis in the simultaneous treatment group. The values of fibrinogen (*p* < 0.001) and D‐dimer (*p* < 0.001) at T0 were significantly different from the values at T1, T2, and T3. (Figure 3).

**TABLE 2:** The differences of coagulation indicators between the simultaneous treatment group and control group at different time points.

	T0		T1		T2		T3	
	Mean difference (SD)^a^	*p* Value	Mean difference (SD)^a^	*p* Value	Mean difference (SD)^a^	*p* Value	Mean difference (SD)^a^	*p* Value
Platelet indicators								
PLT (10^9^/L)	25.8 (30.9)	0.415	25.3 (28.3)	0.384	14.5 (29.0)	0.623	10.1 (26.7)	0.709
PCT (%)	0.02 (0.03)	0.491	0.01 (0.03)	0.521	0.02 (0.03)	0.457	0.02 (0.03)	0.437
MPV (fl)	−0.1 (0.4)	0.682	−0.1 (0.3)	0.886	0.05 (0.4)	0.895	−0.2 (0.4)	0.627
PDW (%)	−0.2 (0.7)	0.814	0.0 (0.6)	1.000	−0.1 (0.7)	0.938	−0.5 (0.7)	0.449
AA (%)	2.4 (12.3)	0.849	−9.0 (12.0)	0.462	−6.8 (12.2)	0.586	−8.3 (10.8)	0.449
ADP (%)	14.1 (9.4)	0.146	17.3 (7.7)	0.040	5.3 (10.6)	0.622	7.9 (9.1)	0.397
Fibrinolysis Indicators								
Fibrinogen (g/L)	0.9 (0.4)	0.038	−0.0 (0.4)	0.928	−1.3 (0.3)	<0.001	−1.5 (0.3)	< 0.001
D‐dimer (μg/mL)	−0.7 (0.8)	0.382	15.3 (1.6)	<0.001	17.1 (1.2)	<0.001	16.9 (1.3)	<0.001
Coagulation indicators								
PT (s)	0.2 (0.3)	0.529	−0.3 (0.4)	0.486	−0.3 (0.7)	0.614	−3.0 (1.6)	0.067
PTA (%)	−1.6 (4.2)	0.706	4.7 (5.2)	0.371	2.1 (6.9)	0.761	16.4 (10.3)	0.121
TT (s)	−0.2 (0.6)	0.780	4.7 (2.1)	0.371	8.1 (2.4)	0.002	4.7 (1.5)	0.004
APTT (s)	−0.5 (1.7)	0.774	−6.0 (5.9)	0.321	−1.9 (2.3)	0.405	−5.7 (3.3)	0.098
INR	0.01 (0.03)	0.655	0.04 (0.05)	0.415	0.04 (0.07)	0.540	−0.34 (0.18)	0.065

*Note*: *p* value less than 0.05 is considered a significant difference. Abbreviations: AA, AA‐induced platelet aggregation rate; ADP, ADP‐induced platelet aggregation rate; APTT, Activated partial thromboplastin time; INR, International normalized ratio; MPV, Mean platelet volume; PCT, Platelet crit; PDW, Platelet volume distribution width; PLT, Platelet count; PT, Prothrombin time; PTA, Prothrombin time activity; TT, Thrombin time.

^a^ Mean difference of the simultaneous group minus the control group.

### 3.3 The effect of batroxobin on coagulation indicators

The results of the paired T‐test showed that batroxobin could significantly affect TT and APTT values in the pre‐treatment group. The TT values significantly prolonged after batroxobin use [T1–T0: 4.0 (7.1), *p* = 0.046; T2–T0: 8.7 (9.3), *p* = 0.003; T3–T0: 5.8 (5.2), *p* < 0.001]. The APTT values greatly increased after batroxobin use [T1–T0: 4.1 (6.1), *p* = 0.021; T2–T0: 5.5 (7.3), *p* = 0.012; T3–T0: 4.3 (6.7), *p* = 0.026] (Table 1). Significant differences were also found in the results of repeated measurement data and interaction effect analysis of TT (*p* = 0.004) and APTT (*p* = 0.053). (Figure 2).

Significant effects of batroxobin on coagulation indicators were observed only in TT values in the simultaneous treatment group. TT values were significantly prolonged at T2 and T3 time points after batroxobin use compared with no batroxobin use. (T2: 8.1 (2.4), *p* = 0.002; T3: 4.7 (1.5), *p* = 0.004). (Table 2) A significant difference was still found in the repeated measurement and interaction effect analysis results in the simultaneous treatment group (*p* = 0.005). (Figure 3).

From the results of the simultaneous treatment group and control group, as shown in the repeated measurements, the use of batroxobin can alleviate the amplitude of changes in the values of coagulation indicators other than TT and exacerbate the amplitude of change in the value of TT caused by anticoagulants. The change trend of PT, PTA, APTT, and INR in the simultaneous treatment group was more gradual, while the change trend of TT was more drastic than that in the control group. (Figure 3).

## 4 DISCUSSION

In this real‐world study, we examined the effects of batroxobin on platelet, fibrinolysis, and coagulation functions in CVT patients under different medication regimens to provide a basis for its safe application in clinical practice.

Batroxobin, known as defibrinase, primarily exerts its pharmacological effects by degrading fibrinogen. Previous studies have shown a rapid decrease in fibrin degradation products, accompanied by a sudden rise in ‐dimer, during batroxobin administration, and our results are consistent with these findings.^11^ Batroxobin also seemed to indirectly affect the coagulation system mainly through the significant decrease of fibrinogen and the increase of fibrin degradation products.

Our results revealed that batroxobin did not affect platelet count and volume but inhibited the ADP‐induced platelet aggregation rate. Platelet aggregation, the process by which platelets adhere to one another, is critical for thrombosis. Upon platelet activation, the GPIIb‐IIIa complex exposes the fibrinogen receptor and binds to it, resulting in aggregation.^16^, ^17^ Fibrinogen is essential for platelet aggregation, and its reduction by batroxobin may inhibit this process.^18^ Additionally, D‐dimer, which can inhibit fibrin formation, has antithrombin effects and inhibits platelet adhesion, aggregation, and release.^19^, ^20^ The elevation of D‐dimer induced by batroxobin may further affect platelet aggregation. Therefore, monitoring platelet function during batroxobin use is essential.

Batroxobin has been evaluated in patients with various conditions, including stroke and CVT. Studies comparing these agents with heparin have shown significantly less bleeding in patients treated with defibrinogenating enzymes than in those treated with heparin.^21^ Moreover, batroxobin combined with anticoagulation did not increase the risk of intracranial hemorrhage compared to anticoagulation alone.^14^, ^22^, ^23^ Our results seem to support these clinical observations. This study showed that, compared to anticoagulant therapy alone, the trend in coagulation indicator values in the batroxobin combined with the anticoagulant group was slower, with no significant differences between two time points. Batroxobin can moderate the changes in coagulation indicator values caused by anticoagulant drugs, allowing its pharmacological effects without causing abrupt changes in the coagulation system or inducing bleeding events, suggesting that batroxobin is a potentially safe and effective adjunct therapeutic agent. Batroxobin's “mitigating effect” might be attributed to the generation of a substantial amount of fibrin degradation products (FDPs) following its administration. These FDPs have a procoagulant effect that may counterbalance the anticoagulant drugs, resulting in a more gradual change in coagulation indicators. However, this mechanism requires further validation through subsequent experiments.^24^

The elevation of D‐dimer and reduction of fibrinogen after batroxobin strongly support endogenous tPA‐promoting thrombolysis. TT reflects the duration required for converting fibrinogen to fibrin, and prolonged TT after batroxobin use indicates fibrinogen deficiency, dysfunction, or consumption. Changes in these parameters following batroxobin treatment further confirm its ability to reduce fibrinogen.

**FIGURE 4:**
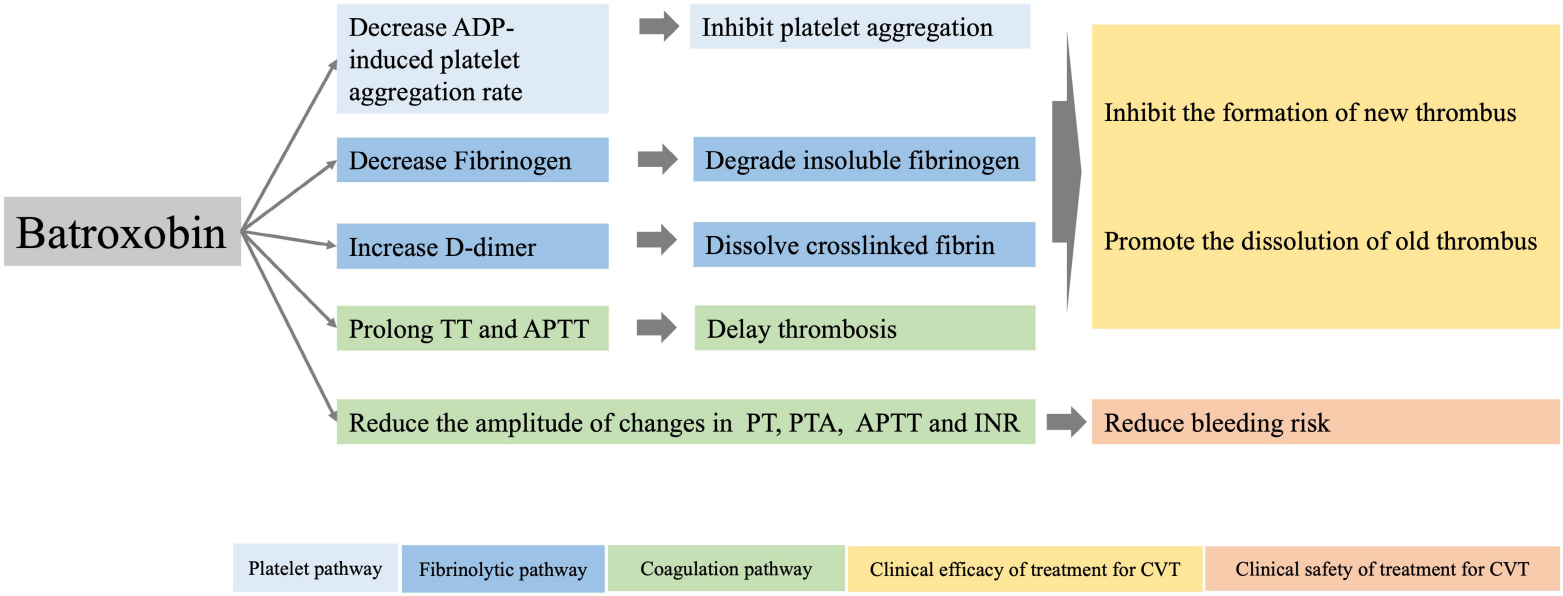
Mechanism diagram of safety and efficacy of batroxobin in treating CVT. APTT, Activated partial thromboplastin time; CVT, Cerebral venous thrombosis; INR, International normalized ratio; PT, Prothrombin time; PTA, Prothrombin time activity; TT, Thrombin time.

In brief, batroxobin can inhibit platelet aggregation, degrade insoluble fibrinogen, dissolve soluble fibrin, and prolong TT and APTT, forming the primary mechanism of its antithrombotic action. It plays a stable role in effectively treating CVT. Additionally, the adjuvantive use of batroxobin can slow down the changes in coagulation indicators caused by anticoagulant drugs, which may be one of the mechanisms why batroxobin combined with anticoagulant therapy does not increase the risk of adverse events, particularly bleeding. However, the interaction effects of the functional changes in each system on the clinical impact after batroxobin use need further clarification. Specifically, does the prolongation of TT and APTT by batroxobin and the delay in other clotting indicators reduce the efficacy of anticoagulants? Can the enhanced antithrombotic ability due to platelet inhibition and fibrinolytic enhancement of batroxobin offset the decreased antithrombotic ability due to coagulation inhibition? These questions need to be further clarified by subsequent high‐quality research. (Figure 4).

### 4.1 Limitations

Limitations of this study include a small sample size, which underpowered the study and restricted its clinical scope; future studies with larger sample sizes are needed to enhance the reliability and persuasiveness of the research. Additionally, as with any retrospective study, the loss of related data is another significant limitation, particularly in rare diseases such as CVT. Furthermore, since CVT requires timely anticoagulant therapy and most patients at our hospital are referred from other facilities, the complexity of prehospitalization medication limits the number of eligible patients included in this study.

## 5 CONCLUSION

Batroxobin can significantly inhibit ADP‐induced platelet aggregation rate, increase D‐dimer, decrease fibrinogen, and prolong TT and APTT when used alongside anticoagulant agents. Additionally, the adjuvant administration of batroxobin can reduce the amplitude of changes in coagulation indicators caused by anticoagulants. This may be one of the mechanisms explaining why batroxobin combined with anticoagulant therapy does not increase the risk of bleeding.

## AUTHOR CONTRIBUTIONS

MR: manuscript drafting and revision, and study concept and design. LD: manuscript drafting and revision, study concept and design, collection, assembly, and interpretation of the data. S‐SY, W‐MQ, Z‐XM, H‐XQ, G‐YB: collection, assembly, and interpretation of the data. MR, LD, J‐BL: manuscript writing and final approval of the manuscript. MR, J‐XM, and D‐YC deeply edited the revised version and contributed to the critical revision.

## FUNDING INFORMATION

This study was sponsored by the National Natural Science Foundation of China (grant number 82171297 and 82101390), and the Beijing Natural Science Foundation (grant number 7212047).

## CONFLICT OF INTEREST STATEMENT

The authors declare that there is no conflict of interest.

## INSTITUTIONAL REVIEW BOARD STATEMENT

Ethical review and approval were waived for this study due to this is retrospective study.

## INFORMED CONSENT STATEMENT

Informed consent was obtained from all subjects involved in the study.

## Supporting information


Table S1.

## Data Availability

The data and materials are available on request to the corresponding author.
