# Supplementary material for: Effects of batroxobin on the antithrombotic system in patients with cerebral venous thrombosis: Clues to mechanisms
Source: CNS Neurosci Ther. 2024 Aug 4;30(8):e14861. doi: 10.1111/cns.14861 (PMC11298196; doi:10.1111/cns.14861)
Supplement: Supplementary file 1 — Table S1. [file CNS-30-e14861-s001.docx]

Supplementary table 1: Demographic and basic clinical features

| **Variables** | **Pre-treatment group** | **Simultaneous treatment group** | **Control group^#^** | **P*** |
| --- | --- | --- | --- | --- |
| **Personal data** |  |  |  |  |
| Age, mean ± SD, years | 43.3±16.5 | 37.0±15.5 | 31.4±10.3 | 0.254 |
| Gender (M: F) | 22：38 | 7：8 | 4：11 | 0.256 |
| **Risk factors, n (%)** |  |  |  |  |
| Hyperlipidemia | 16 (26.7%) | 2 (13.3%) | 0 | 0.143 |
| HBP | 15 (25%) | 1 (6.7%) | 0 | 0.309 |
| Type 2 DM | 4 (6.7%) | 0 | 1 (6.7%) | 0.309 |
| Pregnancy | 2 (3.3%) | 4 (50%) | 5 (45.5%) | 0.690 |
| OCP use | 2 (3.3%) | 1 (6.7%) | 2 (13.3%) | 0.543 |
| DVT | 1 (1.7%) | 2 (13.3%) | 0 | 0.143 |
| Pulmonary embolism | 0 | 1 (6.7%) | 0 | 0.309 |
| Smoking | 3 (5%) | 1 (6.7%) | 0 | 0.309 |
| Drinking | 2 (3.3%) | 1 (6.7%) | 2 (13.3%) | 0.543 |

HBP: High blood pressure; DM: diabetes mellitus; OCP: Oral contraceptive pill; DVT: Deep vein thrombosis.

* P value represents the significance of the difference between simultaneous group and control group. P value less than 0.05 is considered a significant difference and is shown in bold in the table.

^#^ This control group was only contrasted with the simultaneous treatment group in this paper.
